# Molecular Events Involved in Fruitlet Abscission in Litchi

**DOI:** 10.3390/plants9020151

**Published:** 2020-01-24

**Authors:** Minglei Zhao, Jianguo Li

**Affiliations:** 1 State Key Laboratory for Conservation and Utilization of Subtropical Agro-Bioresources, China Litchi Research Center, South China Agricultural University, Guangzhou 510642, China; zml503@scau.edu.cn; 2 Guangdong Litchi Engineering Research Center, College of Horticulture, South China Agricultural University, Guangzhou 510642, China

**Keywords:** *Litchi chinensis* Sonn, fruit abscission, carbohydrate and hormones, LcHB2/3, LcIDL1-LcHSL2

## Abstract

Abscission in plants is an active and highly coordinated physiological process in which organs abscise from the plant body at the abscission zone (AZ) in responding to either developmental or environmental cues. Litchi (*Litchi chinensis* Sonn.) is an important economic fruit crop widely grown in Southeast Asia particularly in South China. However, the excessive fruit drop during fruit development is a major limiting factor for litchi production. Thus, it is an important agricultural concern to understand the mechanisms underlying the fruit abscission in litchi. Here, we present a review focusing on the molecular events involved in the fruitlet abscission. We also highlight the recent advances on genes specifically associated with fruit abscission and perspectives for future research.

## 1. Introduction

Abscission in plants is a process that facilitates the vegetative and reproductive organs to be shed at the abscission zones (AZs) in response to either developmental or environmental cues [1]. As sessile organisms, plants have evolved this highly advantageous process to propagate successfully and respond to biotic and abiotic stresses, as well as nutrient and hormone imbalance. However, in an agricultural perspective, abscission will result in great loss for crop productivity [1]. Thus, it is of great importance to understand deeply the mechanisms underlying abscission. It is widely accepted that abscission is a highly regulated process. Using Arabidopsis as a model plant, several research groups have revealed that the floral organ abscission in Arabidopsis is tightly coordinated by an INFLORESCENCE DEFICIENT IN ABSCISSION (IDA)-HAESA (HAE)/HAESA-like2 (HSL2) kinases signaling module (IDA-HAE/HSL2 signaling module) [2,3,4,5,6,7]. However, whether the IDA-HAE/HSL2 signaling module is conserved in cell separation processes in other species remains poorly understood.

**Figure 1:**
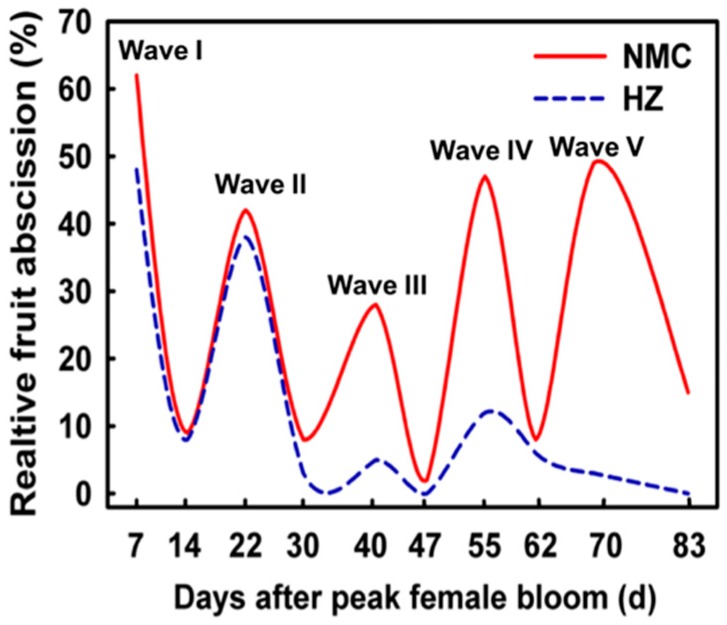
Fruit abscission pattern of litchi for normal-seeded cultivar ‘HZ’ (cv. Huaizhi) and aborted-seeded cultivar ‘NMC’ (cv. Nuomici).

Litchi (*Litchi chinensis* Sonn.) is a tropical and subtropical fruit that originated in Southern China. It has been under cultivation for more than 2300 years in China. Now it is widely cultivated in over 20 countries due to its delicious and nutritive fruits. Litchi industry has great economic value and accounts for a large proportion in the gross agricultural product in many regions. However, litchi suffers from the widespread problem of low bearing. In the period 2013–2015, the average yield of litchi in China was only 3.8 t ha^−1^ [8]. Different factors may be responsible for this poor productivity, of which excessive precocious fruit abscission is the main factor. The litchi ovary is bi-lobed, with one ovule in each lobe. Usually, only one ovary can develop into a fruit and the other one atrophies after pollination and fertilization. There are two distinct fruit growth stages. Stage I lasts for about 53 days (d) after female flower bloom, which is dominated by the growth of pericarp and seed coat. At the end of this stage, the fruitlet weighs about 3–4 g. Stage II is dominated by the growth of embryo and aril, which lasts about 35 d [9]. According to our unpublished results, there are 3–5 fruit drop waves (I, II, III, IV, V) dependent on cultivars (Figure 1) during fruit development about three months after pollination based on the relative abscission rate. Wave I accounts for about 60% of the total fruit drop. In addition to poor pollination and fertilization, it is mainly related to the huge amount of flowers. Generally, it produces hundreds and thousands of flowers per inflorescence. In particular, the large and concentrated blooming of the second male flower (M2) causes a large number of fertilized ovary to drop. M2 flowers might deplete assimilates needed for the fertilized ovaries and removing M2 flowers can significantly increase the initial fruit set [10]. Wave II accounts for about 30–40% of the remaining fruitlet. Carbohydrate stress [11,12,13,14,15,16], high acid level (ABA), and low indole-3-acetic acid (IAA) content in fruit [17,18,19] are considered to be important physiological reasons for this fruit drop wave. Wave III is specific for aborted-seed cultivars such as ‘Nuomici’, accounting for about 20–30% of the remaining fruitlet. The failure of embryo [20,21,22], high ABA content, and low cytokinins (CTKs) and gibberellins (GAs) content in fruit [18,19] are regarded as important physiological reasons for this fruit drop wave. Wave IV is not so obvious in normal-seeded cultivars such as ‘Huaizhi’ than that in aborted-seeded cultivars such as ‘Nuomici’, which accounts for about 10–20% and 20–40% of the remaining fruit, respectively. The rapid growth of aril and embryo, and the summer flush are coincided with this drop wave. The nutrition competition among sinks might contribute to the extent of this drop wave [19,23]. Wave V is called preharvest fruit drop (PFD), which accounts for 20–50% of the remaining fruit. Mitra et al. [24] found that some normal-seeded cultivars had the preharvest drop wave in India. However, in China, we found only some aborted-seeded cultivars (‘Nuomici’, ‘Wuheli’, ‘Xingxinxiangli’, and ‘Jizuili’) had PFD. High ABA and low IAA concentration in seed might be associated with PFD [17,19].

Therefore, the first three waves are the main fruit drop period of litchi, which occurs at stage I of fruit growth, and thus called fruitlet abscission. Fruitlet growth after fertilization requires a continuous supply of carbohydrates provided from current photosynthetic production. When carbon supply is limited, fruit growth rate declines until it reaches an irreversible low level, which stimulates the fruitlet abscission. Generally, it is always with overcast and rainy weather during the fruitlet development of litchi. Artificial shading of litchi leaves to imitate overcast weather or spraying of photosynthetic inhibitors causes serious fruit abscission [17]. Litchi fruitlet, possessing strong sink strength, could mobilize carbohydrate from source leaves in support of normal fruitlet development. However, the photosynthetic efficiency of litchi leaves is very low (about 3 μm CO_2_ m^−1^ s^−1^) compared to high photosynthetic efficiency fruit trees such as apple (about 6–22 μm CO_2_ m^−1^ s^−1^). It is suggested that normal litchi fruit development relies greatly on current carbohydrate reserve in source leaves [11], which means that if much more carbohydrate is available in source leaves the fruit abscission rate will be reduced. Yuan and Huang found that the second to fourth waves of abscission were much less intensive in trunk-girdled ‘Nuomici’ trees at full bloom than that in ungirdled trees, which is primarily due to the new root and shoot growth that was inhibited by girdling treatment, thereby strengthening the fruit in the competition of carbohydrate [12]. As trunk-girlding treatment is carried out at full bloom, the intensity of the first wave of fruitlet abscission was only slightly inhibited, however, it could not exclude that the first wave of abscission in litchi is closely associated with carbohydrate supply. In fact, the consumption of carbohydrate reserve of flowering is higher in ‘Feizixiao’ than in ‘Baitangying’ as ‘Feizixiao’ has significantly larger panicles than ‘Baitangying’, thereby leading to higher fruitlet abscission of the first wave in ‘Feizixiao’ than that in ‘Baitangying’ [25]. Recently, it was shown that girdling plus defoliation treatment (GPD) significantly decreased the soluble sugar content and IAA level, but induced the ethylene production in the fruitlet of litchi [15,16]. Based on these results, we propose that carbohydrate deficiency is likely the first event within abscising fruitlet and acts as an abscission signal perceived by fruitlet, then the endogenous hormones in the fruitlet are changed and transported as a signal to AZ to activate the abscission process in litchi.

In order to get more homogeneous fruitlet and fruitlet abscission zone (FAZ) samples to study the changes in gene expression related to fruitlet abscission in litchi, rapid and highly reproducible abscission-accelerating approaches conducted after wave II of abscission were used in our studies. Carbohydrate stress treatments such as shading, GPD, and ethephon application (ETH) had been proven to be reliable experimental models that induce more than 90% fruitlet to drop within one week [15,17,26,27,28,29]. Whereas the 2,4-dichlorophenoxyacetic acid (2,4-D) was shown to be an effective chemical to reduce the litchi fruitlet abscission [30]. To investigate the molecular events associated with fruitlet abscission under these treatments in litchi, the RNA-seq approach was used to profile the differentially expressed genes (DEGs) (Table 1). Recently, some of these DEGs such as *LcIDL1* and *LcHB2*/*3* have been functionally analysed in the control of abscission, and were regarded as potential candidate genes for further biotechnological applications [26,29,31]. In this review, we mainly highlight current knowledge of molecular aspects and key genes involved in fruitlet abscission in litchi.

## 2. Reduced Lignified Cells at the Fruitlet AZ in Litchi

**Figure 2:**
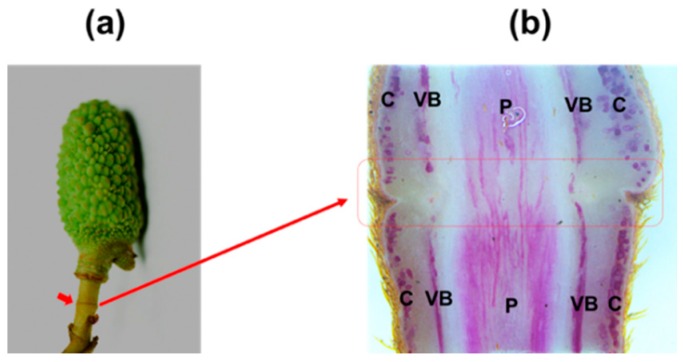
Lignin deposition within the peduncle abscission zone (AZ) in Litchi. (**a**) Fruitlet at 25 days after anthesis. Red arrow indicates the peduncle AZ. (**b**) Lignified cells within peduncle stained with phloroglucinol-hydrogen chloride (HCl). The red box indicates the AZ. Note that the interruption of lignified cells in the cortex, and reduced lignified cells in the pith region. C: Cortex; VB: Vascular bundle; P: Pith region.

The AZ is a specialized tissue that differentiates at a predetermined region on the organ that will shed. Anatomical assays revealed that an AZ consists of several layers of cells, which are clearly distinguishable from their neighbouring cells as they are smaller, with dense cytoplasm and are interconnected by plasmodesmata [32]. AZs differentiate in well-defined positions usually at the boundary between the organ to be shed and the plant body such as the flower AZs in peach [33]. Nevertheless some AZs locate at the proximal or distal end within the pedicel such as the flower pedicel AZ of tomato and citrus plants, respectively [34,35]. In litchi, the differentiation of pedicel AZ is initiated when the flower sepal differentiates from the primordium. At anthesis, the mature pedicel AZ tissues, which locate at the proximal end within the pedicel, have developed into seven to ten cell layers that extend across the pedicel [29]. Soon after successful fertilization, as the peduncle develops the fruit AZ will locate at the mid-peduncle (Figure 2a). Phloroglucinol-HCl staining shows that the intensity of lignin deposition is much less within AZ tissues compared with that within distal or proximal regions (Figure 2b), differently from what is observed at the pedicel AZ in tomato, in which the lignin does not form completely [36]. Lignin is a feature of differentiation in many cell types and the spatial pattern of lignin deposition is closely linked to the function of the cells. Reduced lignin deposition at AZ suggests that AZ maintains meristem-like activity [37,38]. In addition, the role of lignin deposition within AZ has also been suggested to be associated with generating a tension to facilitate cell wall breakage during the fruit abscission in citrus [39].

## 3. The Possible Molecular Events Associated with Fruitlet Abscission within the Fruitlet

Given that carbohydrate deficiency in fruit is likely the first event during the fruit abscission in litchi, the possible molecular events associated with fruit abscission were investigated by RNA-Seq under carbohydrate deficiency-inducing treatments such as shading and GPD [15,16,27,30]. Shading treatment was conducted using a neutral-density black-polypropylene shade cloth allowing 18% of full sun at 30 days after anthesis, while GPD treatment was performed with girdling (a ring of bark about 0.5 cm in width and cambium was removed from the branch base) followed by defoliation (removing all leaves above the girdle) at 35 days after anthesis. It was found that one low-carbohydrate sensitive gene asparagine synthase (AS) was immediately upregulated and was consistently increased in the fruitlet after shading treatment [27]. Meanwhile, the genes encoding enzymes involved in sugar degradation were upregulated, while the genes responsible for sugar synthesis were downregulated in the fruitlet in response to shading or GPD treatment [15,27]. Consistently, the soluble sugar contents (sucrose, glucose, and fructose) were found to be significantly reduced in the fruitlet pericarp and seed of litchi after the GPD treatment [15]. These findings suggest that shading and GPD treatments can induce a transient carbohydrate deficiency stress on the litchi fruitlet development. The changed expression of those genes involved in glycosidases, hydrolases, and transferases was likely a direct or indirect response to this stress, which then aggravated the carbohydrate shortage stress in the fruitlet of litchi. The high expression of carbohydrate metabolism genes was also found in apple after shading treatment [40]. However, sorbitol metabolism seemed to be more important in apple fruitlet drop, which was not found in litchi.

It was suggested that the primary signals triggering the activation of the AZ are generated within the abscising organ. Among these signals, auxin and ethylene are the best candidates due to their high mobility. In litchi, six genes encoding auxin-induced proteins (Aux/IAA) and two genes encoding indole-3-acetic acid-amido synthetase (Gretchen Hagen3, GH3) were immediately downregulated in the fruitlet at two days after the GPD treatment. Five genes encoding auxin response factor transcription factors (ARF) and two genes related to polar auxin influx carrier protein (AUX1/LAX) were also downregulated in fruitlet after GPD treatment [15]. Together, these results, in combination with that the IAA content was reduced in the fruitlet by GPD treatment, suggesting that carbohydrate deficiency not only reduced the IAA content in the abscising fruitlet but also restricted the auxin polar transport to the AZ. Similarly, this hypothesis was supported by the findings in mango fruitlet abscission. In the pericarp of abscising mango fruitlet upon ethephon treatment, two genes related to IAA synthesis (one TRYPTOPHAN AMINOTRANSFERASE RELATED *MiTAR2* and one flavin monooxygenases *MiYUCCA10*), two genes associated with IAA carriers (one PIN-FORMED family of efflux carriers *MiPIN1* and *MiLAX2*), and three MiAux/IAA genes were significantly downregulated [41].

In contrast, ethylene production increased and peaked in GPD-treated fruitlet of litchi, coinciding with the upregulation of genes encoding ethylene biosynthetic enzymes (1-aminocyclopropane-1-carboxylate oxidases *ACO1* and *ACO2*) [15]. Similar results were found in apple fruit [42,43]. According to available data, the role of ethylene in this context seems important. Its gaseous nature likely makes it reach the embryo thereby leading to seed abortion, which then represses the IAA biosynthesis and polar transport. Alternatively, the ethylene generated in abscising organs can move as a gas through the peduncle to the AZ, thereby initiating the abscission process. In apple, it has been hypothesized that an ethylene receptor-based defense system might contribute to protecting central fruitlets from the abscission signal [44]. Within the seed, four ethylene receptor genes including *MdETR1* (ETHYLENE RESISTANCE), *MdETR2*, *MdETR102*, and *MdETR5* are differentially distributed. The ethylene produced by the cortex can diffuse toward the seeds, but once it enters the seed, it is first blocked by the receptors being expressed. Within the lateral fruitlets, the high amount of ethylene coming from the cortex can largely saturate the receptors of the seeds, thereby activating ethylene signaling leading to programmed cell death, embryo abortion, and AZ activation. While the small amount of hormone produced by the cortex of the central fruitlets is not able to saturate the receptors, thus keeping its own signaling blocked and preventing abscission [44]. In the future, it will be of great interest to examine whether this elegant mechanism evolved by seeds to protect themselves from the harmful action of ethylene is conserved during the fruitlet abscission process in litchi.

## 4. The Possible Molecular Events Associated with Fruitlet Abscission within the AZ

As discussed above, the carbohydrate deficiency induced the ethylene production in the fruitlet and restricted the IAA polar transport to the AZ in litchi. So what happened next within the AZ? RNA-seq revealed that 2730 candidate genes in AZ were involved in the litchi fruit abscission process induced by ethephon treatment (Table 1). In agreement with the fact that ethylene operates as an activator, while auxin acts as retardants in abscission [45], 115 out of 195 candidate hormone related genes were involved in biosynthesis and signaling pathway of ethylene and auxin. Among these genes, 47 auxin-related genes including those encoding PIN, AUX1, Aux/IAA, ARF, GH3, and small auxin-upregulated proteins (SAURs) were downregulated within the AZ; while 39 ethylene-related genes including key ethylene biosynthetic genes (*LcACO2*, *LcACO3*, *LcACS1*, *LcACS4,* and *LcACS7*), and ethylene signal pathway related genes such as ethylene receptor *LcETR2*, *LcEBF* (ethylene insensitive 3-binding F-box protein), *LcEIN3*/*EIL* (ethylene insensitive 3 and EIN3-LIKE), and *LcERFs* (ethylene response factors) were upregulated [26,28]. These data, together with the evidence showing that *MdACS5B*, *MdACO*, *MdETR1*, *MdERS1* (ETHYLENE RESPONSE SENSOR 1), and *MdCTR1* (CONSTITUTIVE TRIPLE RESPONSE 1) were found to be increased within the AZ in apple [42,43], support the role of auxin as a negative regulator of AZ cells sensitivity to ethylene’s action [13,14]. Based on these gene expression data, it was suggested that genes involved in auxin transport and signaling are required for the induction of the abscission process during both the early phase within abscising fruitlet and later phase within the AZ in litchi. Moreover, increased ethylene sensitivity within the AZ was possibly a consequence of auxin depletion at the AZ or the ethylene movement from the abscising fruitlet to the AZ through the peduncle tissues, as proposed in a recent review [46]. In contrast, this hypothesis could be also supported by the molecular events found in IAA-treated AZ-A of citrus. Xie et al. showed that three genes including one *PIN*, one *GH3,* and one *Aux*/*IAA* were upregulated at the AZ-A by IAA application. Whereas two *ACO* genes (Cs4g13870 and Cs2g17350) and 12 genes related to ethylene signaling, including *ERF* transcription factor and ethylene receptor were strikingly depressed by IAA, suggesting that IAA in AZ-A could suppress ethylene biosynthesis and signaling, and then inhibit abscission signaling in citrus [47].

Transcription factors (TFs) are concerned as major switches of regulatory cascades during development and various biological processes [48]. RNA-seq showed that a total of 185 different TFs including KNOX (KNOTTED-LIKE HOMEOBOX), HD-ZIP (homeodomain-leucine zipper), bHLH (basic helix-loop-helix protein), NAC (NAM, ATAF1/2, and CUC2), MYB, ARF, ERF, Aux/IAA, WRKY, and LBD were changed within the AZ during the ET-induced fruitlet abscission in litchi [28]. Studies in the model plant Arabidopsis have revealed that three KNOX TFs play critical roles in the control of floral organ abscission [4]. Thus, the KNOX TFs differentially expressed at the AZ of litchi likely play key roles in the control of fruitlet abscission. In addition, the role of both ERF and ARF TFs in the regulation of abscission in tomato and Arabidopsis, respectively, has also been documented [49,50,51]. Interestingly, LcARF5A/B have been suggested to be positively involved in the fruitlet abscission in litchi [52]. Overall, more attention should be paid on these TFs in the future.

## 5. HD-Zip Family Transcription Factors LcHB2/3 Are Involved in the Fruitlet Abscission in Litchi

The loss of organs is achieved by dissolution of the middle lamella and cell walls of the AZ cells, which is hydrolysed by cell wall remodelling enzymes, including endo-(1,4)-β-D-glucanases (or cellulases, CELs), polygalacturonases (PGs), and xyloglucan endotransglucosylase/hydrolase (XTHs) [53,54,55,56,57]. However, how the abscission-related cell wall remodelling genes are regulated remains poorly understood. In litchi, during the GPD/ethephon (ET)-induced fruitlet abscission, cellulase and polygalacturonases activities are increased, pectic polysaccharides and cellulose contents are degraded accordingly at the AZ [26,29]. Further investigation found that the expression of *LcCEL2*/*8* and *LcPG1*/*2*, which are specifically expressed at the AZ, is strongly associated with the fruitlet abscission in litchi. Moreover, the promoters of *LcCEL2*/*8* contain the *cis* elements responsible for HD-Zip TF binding, and LcHB2 has been identified to bind directly to the promoters of *LcCEL2*/*8* and activate their transcription. In fact, *LcHB2* is upregulated specifically at the AZ during the GPD/ET-induced fruitlet abscission in litchi [29]. HD-Zip family genes are a class of plant-specific transcription factors (TFs) that consist of a homeodomain (HD) and a leucine zipper (LZ) motif [58]. The HD-Zip TFs mediate genes transcription by binding to target genes promoter via the HD domain, thereby playing specific roles in various plant development aspects [59]; however, whether HD-Zip TFs are involved in the shedding of plant organs has not yet been studied in other plant species. Thus, these findings in litchi provide new information regarding the transcriptional regulation of the cell wall genes involved in plant organ shedding. In addition, *LcPG1*/*2* should be regulated by other transcription factors other than HD-Zip TF as the promoters of *LcPG1*/*2* have no HD binding sites.

Moreover, electrophoretic mobility shift assays and transient expression experiments demonstrated that both LcHB2 and LcHB3 can directly bind to the promoter of *LcNCED3,* one gene encoding 9-cis-epoxycarotenoid dioxygenase (NCED) critical for ABA biosynthesis, *LcACO2/3*, and *LcACS1/4/7* genes and activate their expression. These findings suggest that LcHB2/3 are involved in the fruitlet abscission in litchi by coordinating genes associated with both hormone and cell wall metabolism. 

## 6. The Involvement of IDA-HAE/HSL2 Signaling Module in the Fruitlet Abscission in Litchi

A milestone in abscission research was the identification of the *IDA* in Arabidopsis as mutation in such gene blocks floral organ abscission completely [2]. *IDA* encodes a small signal peptide that is perceived by the leucine-rich repeat receptor-like kinases HAESA (HAE) and HAE-LIKE2 (HSL2) [60]. In litchi, three IDA-like homologs were identified in the genome. It was shown that the closest IDA homolog *LcIDL1* was significantly increased at the AZ during the GPD/ET-induced fruitlet abscission. Further investigation suggested that LcIDL1 largely plays a critical role in inducing the fruitlet abscission in litchi since its ectopic expression in both Arabidopsis wildtype and *ida* background promoted the floral organ abscission [31]. More recently, an HAESA-like homolog, *LcHSL2*, was also isolated from the litchi genome. Though ectopic expression of *LcHSL2* in wildtype Arabidopsis had no effect on the floral organ abscission, its presence in the *hae hsl2* mutant background completely restored the floral organ abscission. Same as *LcIDL1*, the expression level of *LcHSL2* was upregulated during ET-induced fruitlet abscission in litchi [61]. These results suggest that the IDA-HAE/HSL2 signaling module is likely conserved during the fruitlet abscission process in litchi. However, whether LcIDL1 and LcHSL2 could form a ligand-receptor complex requires further investigation, which can be done primarily through an ox-burst system established by Butenko lab [60]. In fact, recent studies have shown that IDA-HAE/HSL2 signaling components were found across the plant kingdom, such as citrus, tomato, soybean, oil palm, and poplar [4,62,63,64,65], suggesting that different cell separation processes might share the IDA-HAE/HSL2 signaling module that is conserved across plant species.

## 7. Conclusions and Future Perspectives

**Figure 3:**
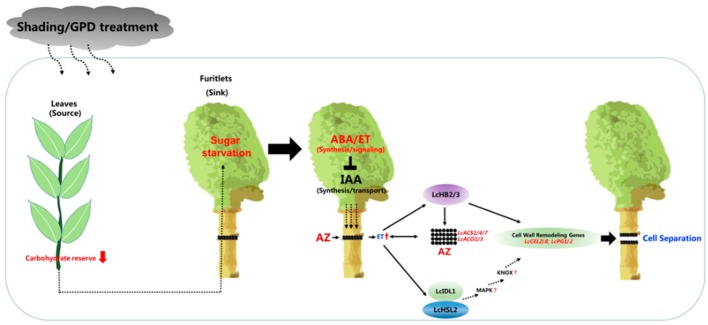
Schematic representation of current understanding on molecular events involved in the fruitlet abscission in litchi.

Taking into account the overall transcriptomic data and some key genes proved to be closely involved in the abscission process, the current understanding of the induction of fruitlet abscission in litchi can be summarized in Figure 3. Based on this model, litchi fruitlet abscission takes place in three main steps, corresponding to the three structural levels where the key events may occur (the bearing shoot, the fruitlet, and the abscission zone). The initial steps occur at the bearing shoot level, where a reduced carbohydrate reserve is established in source leaves either naturally or upon shading/GPD treatments. Since litchi fruitlet development relies greatly on current carbohydrate reserve in source leaves [11], the leaves of bearing shoot are not capable of supporting all the growing fruitlets, leading to the “weaker” ones to drop as they are with lower sink activity. At this stage, how is this nutritional signal transduced into the abscission signal?

Our transcriptomic data suggest that sugar starvation within the fruitlet enhances the ABA synthesis and stimulates the ethylene (ET) production, thereby repressing the IAA synthesis and restricting the polar auxin transport from the fruitlet to the AZ as well. Auxin depletion stimulates the ethylene biosynthesis within the AZ probably by such as LcHB2/3 transcription factors mediating positive regulation of *LcACS1*/*4*/*7* and *LcACO2*/*3*. When the ethylene amount within the AZ is sufficient, it activates the ethylene downstream targets, such as the LcIDL1-LcHSL2 pathway, subsequent activation of cell wall remodelling genes at the AZ, such as *LcCEL2*/*8* and *LcPG1*/*2*, and finally occurrence of fruit abscission. In addition, LcHB2-mediated transcription regulation of *LcCEL2*/*8* could be either LcIDL1-LcHSL2 pathway dependent or not.

Over the past decades, our understanding of the fruit abscission in litchi has progressed rapidly thanks to a combination of transcriptomic and genomic approaches. However, many key questions remain to be answered, for example:

I. To validate whether the activation of AZ is remotely controlled by the fruit as the abscission signals were presumably transmitted from the fruit organ to the AZ through the peduncle.
II. To further investigate whether the IDA-HAE/HSL2 pathway is conserved in litchi since key components of this pathway such as SOMATIC EMBRYOGENESIS RECEPTOR KINASES (SERKs), mitogen-activated protein kinase (MAPKs), and KNOTTED1-LIKE HOMEODOMAIN proteins (KNOXs) have not been identified yet (Figure 2).
III. To elucidate whether a direct link exists between the ethylene signaling and the activation of LcIDL1-LcHSL2 pathway. In fact, that IDA-HAE/HSL2 module can act downstream of ethylene signaling in control of abscission has been suggested not only in litchi but also in other plant species such as tomato, soybean, and oil palm.

First, a reduced carbohydrate reserve is established in source leaves either naturally or upon shading/GPD treatments, leading to the “weaker” fruitlet with sugar starvation as they are with lower sink activity. Then, sugar starvation might serve as a signal to enhance the ABA synthesis and stimulate the ethylene (ET) production, thereby repressing the IAA synthesis and restricting the polar auxin transport from the fruit to the AZ as well. Auxin depletion stimulates the ethylene production within the AZ probably by such as LcHB2/3 transcription factors mediating positive regulation of *LcACS1*/*4*/*7* and *LcACO2*/*3*. *LcHB2*/*3* are expressed at the AZ and are upregulated by ET, thereby enhancing the ET production. When the ethylene amount within the AZ is sufficient, it activates the ethylene downstream targets, such as the LcIDL1-LcHSL2 pathway, subsequent activation of cell wall remodeling genes at the AZ, such as *LcCEL2*/*8* and *LcPG*/*2*, and finally occurrence of fruit abscission. In addition, LcHB2/3 are involved in the fruit abscission via directly promoting the expression of *LcCEL2*/*8*.

## Figures and Tables

**Table 1:** A brief summary of three RNA-seq of the fruitlet abscission in litchi.

Fruitlet Age	Treatments	Tissue Examined	DEGs Number
30 dpa	Shading	Fruitlet	1039
35 dpa	GPD	Fruitlet/AZ	2771
25 dpa	ET	AZ	2730

dpa: Days post anthesis; GPD: Girdling plus defoliation; ET: Ethephon; DEG: Differentially expressed genes.
